# Formation mechanisms for the dominant kinks with different angles in InP nanowires

**DOI:** 10.1186/1556-276X-9-211

**Published:** 2014-05-05

**Authors:** Minghuan Zhang, Fengyun Wang, Chao Wang, Yiqian Wang, SenPo Yip, Johnny C Ho

**Affiliations:** 1 The Cultivation Base for State Key Laboratory, Qingdao University, No. 308, Ningxia Road, Qingdao 266071, People's Republic of China; 2 College of Chemistry and Chemical Engineering, Qingdao University, No. 308, Ningxia Road, Qingdao 266071, People's Republic of China; 3 College of Physics Science, Qingdao University, No. 308, Ningxia Road, Qingdao 266071, People's Republic of China; 4 Department of Physics and Materials Science, City University of Hong Kong, 83 Tat Chee Avenue, Kowloon, Hong Kong

**Keywords:** InP nanowires, Kinks, Microstructures, Formation mechanism, HRTEM, 81.07.-b, 81.05.Ea, 81.07.Gf

## Abstract

The morphologies and microstructures of kinked InP nanowires (NWs) prepared by solid-source chemical vapor deposition method were examined using scanning electron microscopy (SEM) and high-resolution transmission electron microscopy (HRTEM). Statistical analysis and structural characterization reveal that four different kinds of kinks are dominant in the grown InP NWs with a bending angle of approximately 70°, 90°, 110°, and 170°, respectively. The formation mechanisms of these kinks are discussed. Specifically, the existence of kinks with bending angles of approximately 70° and 110° are mainly attributed to the occurrence of stacking faults and nanotwins in the NWs, which could easily form by the glide of {111} planes, while approximately 90° kinks result from the local amorphorization of InP NWs. Also, approximately 170° kinks are mainly caused by small-angle boundaries, where the insertion of extra atomic planes could make the NWs slightly bent. In addition, multiple kinks with various angles are also observed. Importantly, all these results are beneficial to understand the formation mechanisms of kinks in compound semiconductor NWs, which could guide the design of nanostructured materials, morphologies, microstructures, and/or enhanced mechanical properties.

## Background

Recently, III-V compound semiconductor nanowires (NWs), especially InP NWs, have attracted enormous attention in next-generation electronics, sensors, photonics, and solar cells due to their superior carrier mobilities and as direct and suitable bandgaps for efficient photon coupling [1-6]. In the past decade, most studies have focused on understanding and controlling the morphology and structures of InP NWs, such as tapering, reversible switching of growth direction, twin-plane superlattices, and structural transition [2-4,7-11]. However, to the best of our knowledge, few reports are relevant to the kinked InP NWs, particularly the detailed microstructures related to the bending configuration. Generally, it is believed that the kinks in the NWs would influence their transport properties, electron, and hole collection efficiencies for technological applications [12,13]. In this regard, a detailed study on the formation of these kinks is extremely important, which could provide valuable information to further design NW materials with different shapes, morphologies, and microstructures, expanding their application domains [14].

In our experiment, kinked InP NWs frequently emerged in the growing process, which possess a crystal structure of face-centered cubic (zinc blende) [6]. In order to understand the growth mechanism of these bending InP NWs, the morphologies and microstructures of different InP NWs were studied utilizing scanning electron microscopy (SEM) and high-resolution transmission electronic microscopy (HRTEM), respectively. Through comprehensive statistical analysis and intensive structural characterization, it is revealed that the dominant bending angles of InP NWs are approximately 70°, 90°, 110°, and 170°. The formation of bending angles of approximately 70° and 110° is mainly attributed to the occurrence of nanotwins and stacking faults (SFs), which could easily form by the glide of {111} planes. However, for approximately 90° bending, local amorphorization is believed to be the main cause for this phenomenon while approximately 170° kinks are mostly induced by small-angle boundaries, where the insertion of extra atomic planes could make the NWs slightly bent. In addition, NWs with multiple curves composed of different bending angles are also observed.

## Methods

### Synthesis of InP NWs

InP NWs used in this study were prepared by a solid-source catalytic chemical vapor deposition method in a dual-zone horizontal tube furnace as previously reported [6]. Briefly, the solid source (1 g, InP powder, 99.9999% purity) was placed in a boron nitride crucible and evaporated at the center of the upstream zone, while the growth substrate (0.5 nm Au film deposited on SiO_2_/Si) was placed in the middle of the downstream zone with a tilt angle of approximately 20° and a distance of 10 cm away from the source. Au films with a thickness of 0.5 nm were thermally evaporated under a vacuum of approximately 1 × 10^−6^ Torr onto the substrates. During the growth of NWs, the substrate was thermally annealed at 800°C for 10 min in a hydrogen environment (99.999% pure H_2_, 100 sccm, 1 Torr) to obtain Au nanoclusters which acted as the catalysts. When the substrate temperature was cooled to the preset growth temperature (460°C), the source was heated to the required source temperature (770°C) for 60 min. After the growth, the source and substrate heater were stopped and cooled down to the room temperature under the flow of H_2_ gas.

### Characterization of InP NWs

Philips XL30 SEM (Philips, Amsterdam, Netherlands) was employed to view surface morphologies of the grown InP NWs. Selected-area electron diffraction (SAED), bright field (BF) transmission electron microscopy (TEM), and HRTEM were carried out to determine crystal structure and to examine microstructures of the grown InP NWs using a JEOL JEM2100F TEM (JEOL Ltd., Tokyo, Japan) operating at 200 kV. The incident electron beam was along the $\left[1\bar{1}0\right]$ direction. Specimens for HRTEM examinations were prepared by peeling off the InP NWs from the surface of the substrate, ultrasonicating them into anhydrous ethanol for several seconds, and dispersing the finished solution onto a holey-carbon-film-coated copper grid.

## Results and discussion

**Figure 1:**
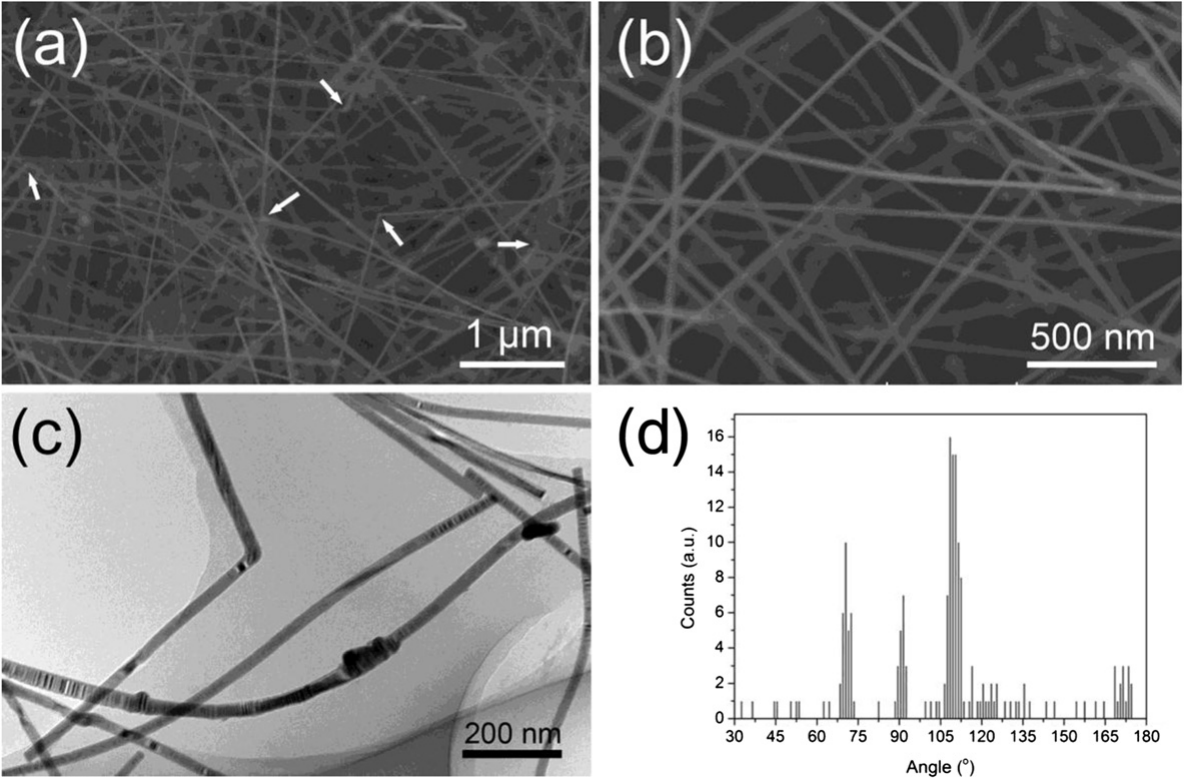
**SEM images depict the morphology of InP NWs along with the statistical graph of kinks. (a)** Low-magnification SEM image of InP NWs prepared by 0.5-nm-thick Au film. Kinks with different angles are clearly observed. Approximately 110° kinks indicated by white arrows show frequently. **(b)** Magnified SEM image shows clear morphology of InP NWs. **(c)** Typical BF image for angle distribution statistic. **(d)** Kink angle statistics of grown InP NWs observed by TEM images. Four dominant groups of kinks with angles of approximately 70°, 90°, 110°, and 170° are clearly displayed.

Figure 1a shows a low-magnification SEM image of InP NWs prepared by 0.5-nm-thick Au catalyst film. It is observed that different kinds of kinks exist in the grown InP NWs. Interestingly, in most cases the bending angles are close to approximately 110° as indicated by white arrows. Magnified SEM image (Figure 1b) exhibits a clear morphology of InP NWs. It is shown that despite there exists some kinks, the overall morphology of grown InP NWs is relatively straight and smooth. As observed from TEM images, InP NWs with kinks can be clearly seen and the diameter of InP NWs is uniform and ranges from 20 to 40 nm. In order to systematically understand the characteristics of the different kinks, initially, a comprehensive statistical analysis was carried out using typical BF TEM images (Figure 1c), which are mainly concentrated in the range of 20 to 30 nm. In this work, the bending angles of more than 180 kinks in different NWs were measured and the statistical result is presented in Figure 1d. It is noted that the angles and frequency of kinks are found independent of the nanowire diameter and four dominant groups of kinks with the bending angles of approximately 70°, 90°, 110°, and 170° are clearly displayed, with the relative percentage of them observed being 17%, 11%, 35%, and 6%, respectively. Except the four dominant groups, the kinked InP NWs with other angles are scarce. Furthermore, the bending angles less than 30° are not observed.

**Figure 2:**
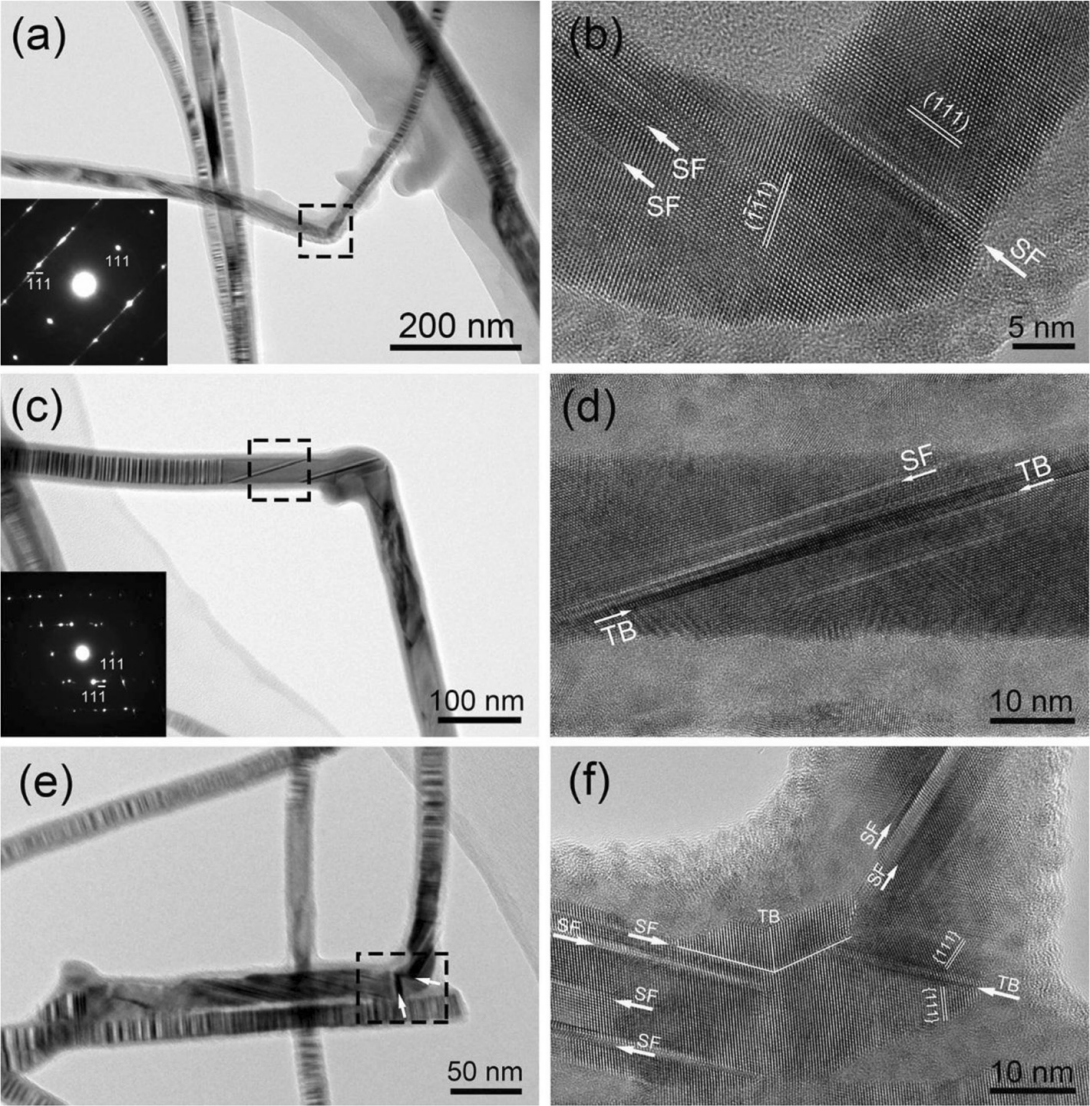
**BF and HRTEM images of approximately 110° kinks in different NWs. (a, c, e)** BF images of 110° kinks. Insets in **(a)** and **(c)** are SAED patterns corresponding to the selected areas. Clear contrast changes are indicated by white arrows in **(e)**. **(b, d, f)** are HRTEM images corresponding to the selected areas in **(a)**, **(c)**, and **(e)** separately. SFs are observed in the kink area in **(b)**. In **(d)**, SFs and twins are shown in the adjacent region to the kink. Large numbers of SFs are observed along the growth direction shown in **(f)**, while twins were observed in the kink area.

To shed light in exploring the formation mechanism of these kinks with different angles, HRTEM technique was exploited to examine the microstructures of these grown InP NWs. Figure 2 shows the typical BF and HRTEM images of approximately 110° kinks observed in different NWs. It is clear that alternating bright/dark contrast appears in a periodic manner along the axial direction of the wire in BF TEM images (Figure 2a,c,e), which indicates the existence of planar defect structure. The phenomenon is consistent with the previous report that high density of SFs in <111> -oriented nanowires commonly form perpendicularly to the growth direction [15]. HRTEM images (Figure 2b,d,f) and corresponding SAED patterns were acquired from the bending areas, which present explicit illustrations of the microstructures in these kink areas. The SAED patterns (Figure 2a,c insets) show the crystal structure of InP NWs here being face-centered cubic (zinc blende). In Figure 2b, it is obvious that the NWs grows along <111> directions and the bending angle is consistent with that between (111) and $\left(\bar{1}\bar{1}1\right)$ planes, namely, approximately 110°. Since the {111} planes are the faces with lower energy in the face-centered cubic structure, the growth of NWs through {111} planes is energetically favorable. Figure 2b also reveals a stacking fault, almost transecting the entire nanowire in the kink area. We suppose that the transecting SFs in the kinked area would be beneficial to the change of growth direction. In addition, nanotwins and SFs were also observed in the region close to approximately 110° kink as depicted in Figure 2d, which corresponds to the selected area in Figure 2c. As mentioned in the previous report [16], the bending of nanowires typically associated with a significantly large local strain in which SFs are induced and resulted to releasing the stress. It is as well noted that an approximately 110° kink consisted of successive curves is observed in Figure 2e. Noticeable contrast variations indicated by white arrows in Figure 2e are supposed to be imaging effects which occur when twin boundary relaxations are present, although it should be pointed out that images with similar appearances could result from astigmatism or misalignment [17]. HRTEM image corresponding to the selected area in Figure 2e is presented in Figure 2f. It is obvious that there is large amount of SFs in the region of approximately 110° kink. In this case, we believe that the larger local strain could be introduced by two successive curves in such narrow space. It is noted that most SFs in the kinked area run nearly parallel to the growth direction. We suppose that in the kinked area, a large amount of stress is introduced such that the {111} planes nearly parallel to the growth direction can easily glide and could facilitate the formation of SFs, which plays an important role in releasing the stress. In addition, nanotwins marked by TB are observed in the bending area. According to the literature, twin-plane formation in zinc blende crystals requires very little energy [18]. The twins are as expected for bulk zinc blende crystals, which can twin on {111} planes by rotating through 60° about the <111> axis [19]. Consequently, in theory, nanotwins could facilitate the NWs to switch from one favorable growth direction to another.

**Figure 3:**
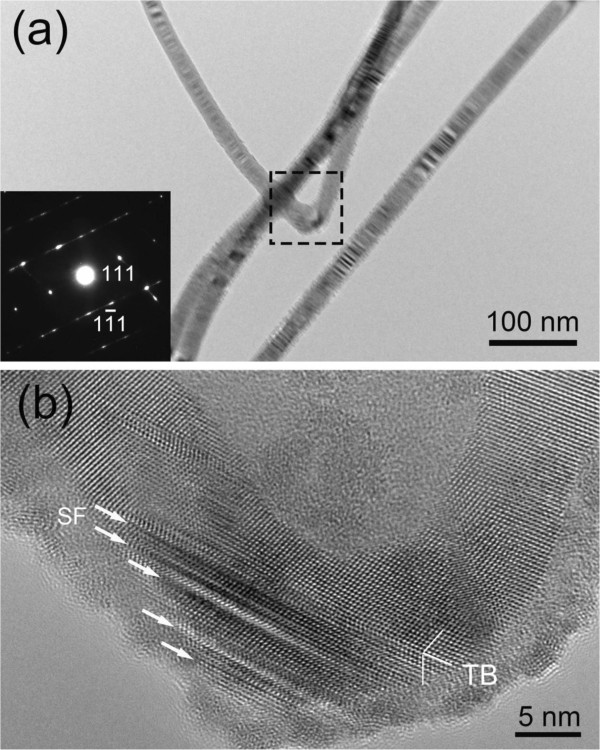
**BF image with corresponding SAED pattern and HRTEM image of approximately 70° kink in InP NWs. (a)** BF image of approximately 70° kink in InP NWs. The SAED pattern from the kink area (inset) matches with cubic zinc blende structure. **(b)** HRTEM image of the selected region in **(a)**. Dense SFs indicated by white arrows emerge in the kink area. The twin indicated by TB appears in the kink area.

Compared with approximately 110° kinks, the approximately 70° kink bends sharply as shown in Figure 3a. Its corresponding SAED pattern (inset) matches well with cubic zinc blende structure, and the lattice planes are {111} planes. As shown in Figure 3b, the nanotwin appears in the bending area, which is similar to that occurs in approximately 110° kinks. As mentioned above, the formation of nanotwin could be beneficial to the change of growth direction. In addition, it is worth noting that highly dense SFs are also observed in the approximately 70° kink area and nearly parallel to the growth direction. In such a sharp bending, the strain is so severe, which could produce the internal stress larger than that in approximately 110° kink.

On the basis of the above observed results, approximately 70° and 110° kinks are believed to form by the glide of {111} planes, which produces nanotwins and SFs to facilitate the formation of such kinks. It is known that {111} planes are the closest packed planes with the lower interfacial energy in cubic zinc blende structure and the angles between two different {111} planes are 70.5° or 109.5°. Therefore, the change of growth direction is inclined to be <111> and the bending angle is mostly close to 70.5° or 109.5°. However, due to their difference in the bending degree, the densities of SFs in local areas for approximately 70° and 110° kinks are different. When the bending angle is approximately 70°, the curvature is so sharp and supposed to cost larger energy. As a result, the internal stress would be larger than that of approximately 110° kinks, which needs massive and dense SFs to release. In addition, the sharp curvature makes the formation of approximately 70° kinks more difficult, which can be interpreted by presence of a smaller percentage with approximately 70° kink than that of approximately 110° kink as illustrated in Figure 1d.

**Figure 4:**
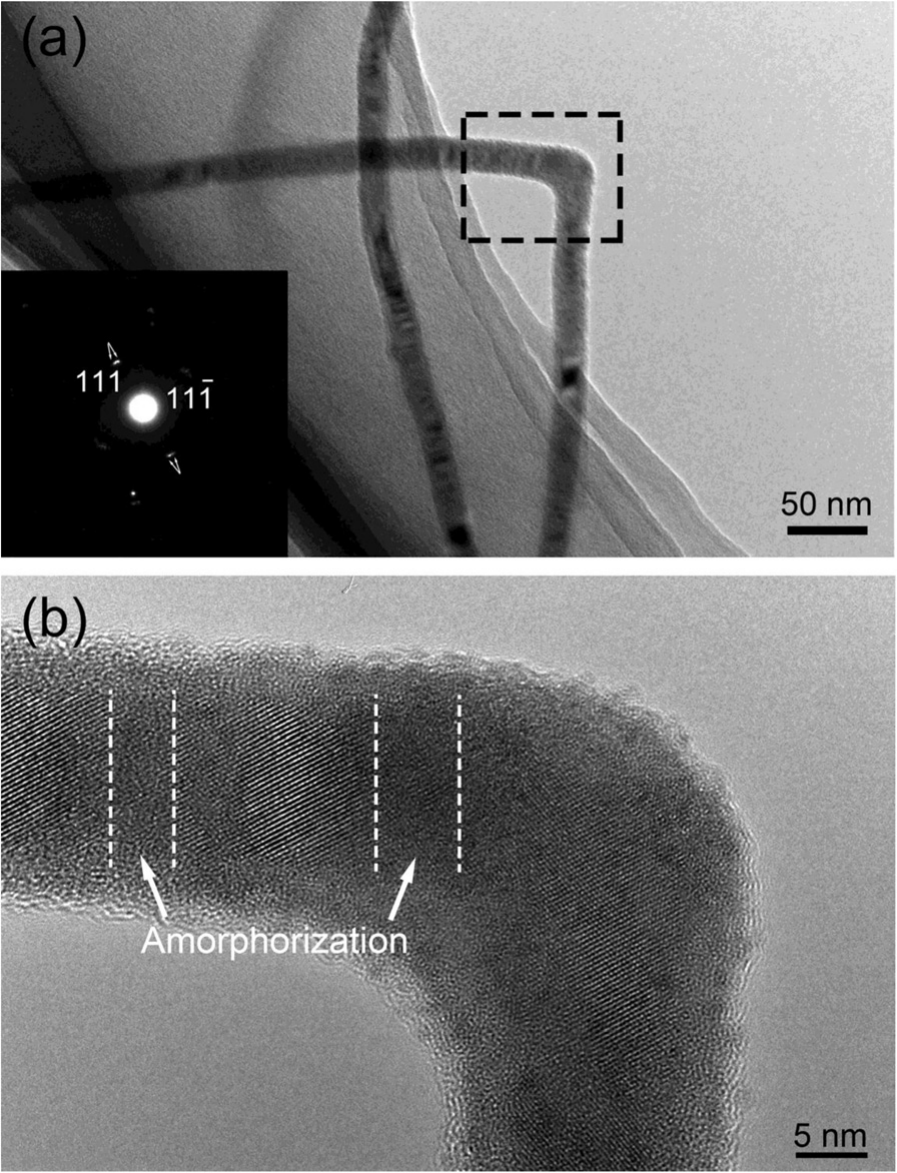
**BF image with corresponding SAED pattern and HRTEM image of approximately 90° kink in InP NWs. (a)** BF image of the kink of approximately 90° in InP NWs. The inset is SAED pattern corresponding to the kink in which the diffraction spots indicated by white arrows are split into irregular spots. **(b)** HRTEM image of the selected area in **(a)**. The observed amorphous regions are pointed by arrows.

In the previous studies, SFs and nanotwins are frequently observed in III-V semiconductor NWs [7,9,20]. Here, the intensive study of microstructures reveals some novel characteristics in the remaining two groups of kinks in InP NWs, i.e., approximately 90° kinks and 170° kinks. As presented in Figure 4a, an approximately 90° kink can be clearly observed. The inset gives its corresponding SAED pattern, in which each diffraction spot indicated by white arrows was split into adjacent irregular spots. It indicates that the crystal orientation makes slight changes in this area. It is evidenced in Figure 4b that the amorphous regions pointed by arrows are firstly observed in the approximately 90° kink, where the crystal orientation is disordered. This result could guide us presenting reasonable explanations for the formation of approximately 90° kinks. In crystallography, it is not easier to form an approximately 90° angle by the glide of {111} planes. Therefore, in order to produce such shape, the change of crystal lattice becomes reasonable. It is known that amorphorization could distort the crystal lattice and break the barrier for the transition of morphology in the growing process. As a result, the growth of NWs would become more flexible, which is beneficial to the formation of approximately 90° kinks.

**Figure 5:**
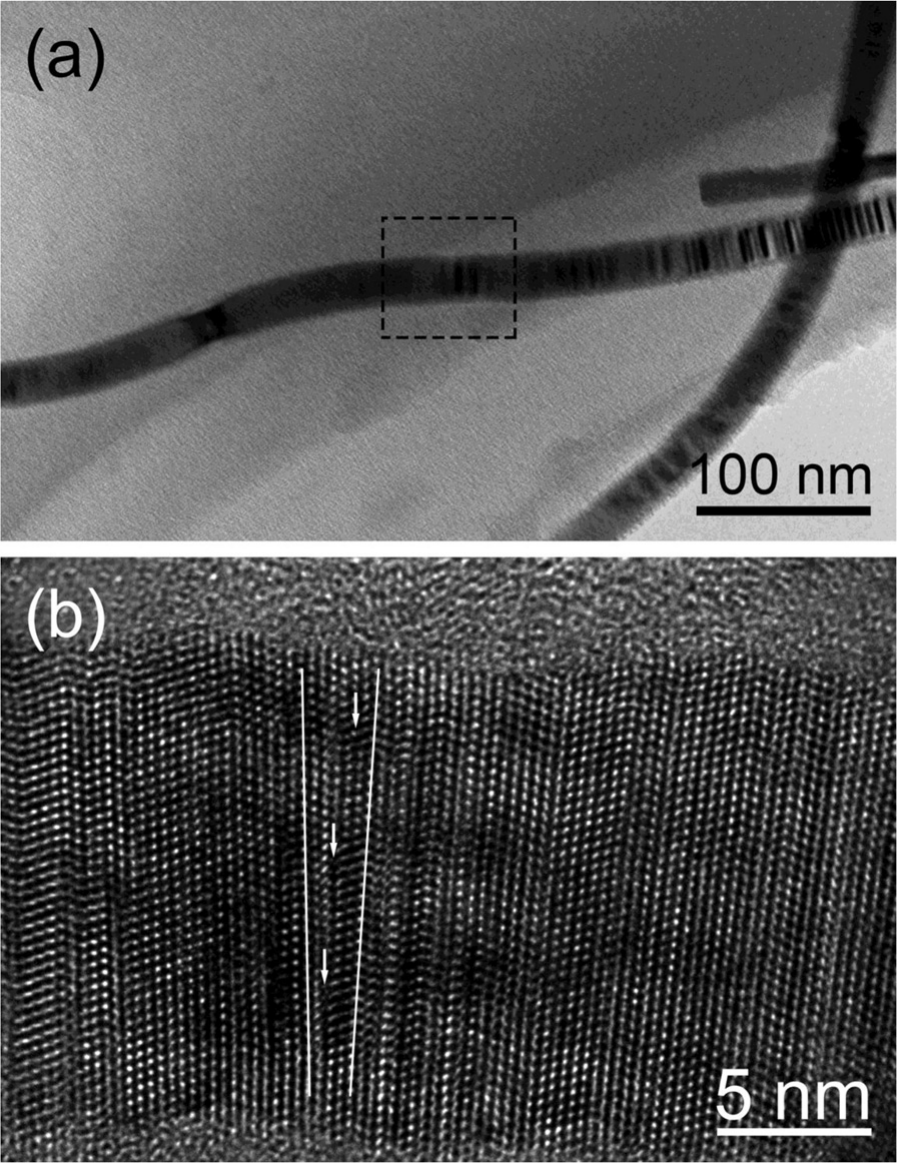
**BF and HRTEM images of approximately 170° kink in InP NWs. (a)** BF image of slight bending InP nanowire, whose bending angel is approximately 170°. **(b)** HRTEM image of the local part selected in **(a)** in which a small-angle boundary is observed.

As for the slight bendings, i.e., approximately 170° kinks, careful examinations show that the small-angle boundary exists in the bending area, being rarely observed in III-V semiconductor NWs [16]. As depicted in Figure 5a, the InP NWs are slightly bent in which planar defects could be easily observed. Furthermore, as given in Figure 5b, a small-angle boundary was clearly seen in the selected area of Figure 5a. The extra atomic planes are inserted as indicated by arrows. This result is similar to that observed in Au NWs [21]. In the growing process, the NWs are likely to be affected by the disturbance of growth conditions, such as the gas flow fluctuation. As a result, the atomic arrangement is likely to collapse and tend to reconstruct in order to accommodate the disturbance effect, which causes the formation of small-angle boundary. The inserted extra atomic planes could generate unbalanced internal stress for the growth of the upper side and lower side of InP NWs shown in Figure 5b. Consequently, the InP NWs show slight bending. In addition, depending on the simulation of Cao et al. [22], the motion of dislocations along the well-defined slip systems can be restricted by twin boundaries (TBs). As observed, there are plenty of TBs in the left and right sides of the small-angle boundary, which would restrict the occurrence and the motion of dislocations. Therefore, the number of kinks with approximately 170° is relatively small.

**Figure 6:**
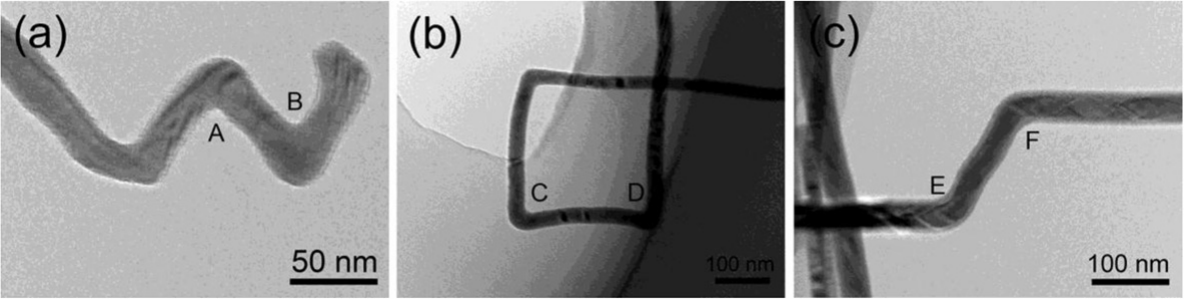
**Various shapes composed of multiple kinks with different angles. (a)** Zig-zag InP NWs composed of three approximately 70° kinks. **(b)** Rectangular InP NWs composed of three approximately 90° kinks. **(c)** InP NWs with two approximately 110° kinks.

In addition to individual kinks, multiple kinks are also frequently observed in InP NWs. As shown in Figure 6, different shapes, such as zig-zag and rectangle, are composed of kinks with different angles mentioned above. They are likely to be formed by the change of growth conditions. At the same time, it is observed that the formation of kinks is not related to the substrate tilting during the growth. For the growth substrate without any tilt angle, the InP NWs with kinks were also frequently observed. The occurrence of continuous kinks means that there is a possibility to produce NWs with different shapes in large scale, such as the nanospring produced in ZnO NWs [16]. Our results also call into question how to control the shape and microstructures of NWs by tuning the NW growth conditions in order to satisfy the needs of practical applications.

## Conclusions

In conclusion, four dominant kinds of kinks with an angle of approximately 70°, 90°, 110°, and 170° have been observed in InP NWs. The dominant InP crystal structure in this work is zinc blende and the kinks with bending angles of approximately 70° and 110° are mainly attributed to the SFs and nanotwins, which could easily form by the glide of {111} planes. However, the approximately 90° kinks result from the local amorphorization of InP NWs while the approximately 170° kinks are mainly caused by small-angle boundaries, where the insertion of extra atomic planes could make the NWs slightly bend. In addition, NWs with multiple kinks in various angles are also observed.

## Abbreviations

BF: Bright field; HRTEM: high-resolution transmission electronic microscopy; NWs: nanowires; SAED: selected-area electron diffraction; SEM: scanning electron microscopy; SFs: stacking faults; TBs: twin boundaries.

## Competing interests

The authors declare that they have no competing interests.

## Authors’ contributions

MHZ analyzed the experimental results and drafted the manuscript. FYW performed the SEM observations and revised the manuscript. CW performed the HRTEM observations. YQW proposed the formation mechanism of the kinks in InP NWs and revised the manuscript. SPY and FYW fabricated InP NWs. JCH directed the experiment of fabricating InP NWs. All authors read and approved the final manuscript.

## References

[B1] Duan X Huang Y Cui Y Wang J Lieber CM Indium phosphide nanowires as building blocks for nanoscale electronic and optoelectronic devices Nature 2001 9 66 69 10.1038/35051047 11343112

[B2] Han N Wang F Hou JJ Yip SP Lin H Xiu F Fang M Yang Z Shi X Dong G Hung TF Ho JC Tunable electronic transport properties of metal-cluster-decorated III-V nanowire transistors Adv Mater 2013 9 4445 4451 10.1002/adma.201301362 23784849

[B3] Johansson J Karlsson LS Svensson CP Martensson T Wacaser BA Deppert K Samuelson L Seifert W Structural properties of <111> B-oriented III-V nanowires Nat Mater 2006 9 574 580 10.1038/nmat1677 16783358

[B4] Caroff P Dick KA Johansson J Messing ME Deppert K Samuelson L Controlled polytypic and twin-plane superlattices in III-V nanowires Nat Nanotechnol 2009 9 50 55 10.1038/nnano.2008.359 19119283

[B5] Han N Hou JJ Wang F Yip S Yen YT Yang ZX Dong G Hung T Chueh YL Ho JC GaAs nanowires: from manipulation of defect formation to controllable electronic transport properties ACS Nano 2013 9 9138 9146 10.1021/nn403767j 24016352

[B6] Hui AT Wang F Han N Yip S Xiu F Hou JJ Yen YT Hung T Chueh YL Ho JC High-performance indium phosphide nanowires synthesized on amorphous substrates: from formation mechanism to optical and electrical transport measurements J Mater Chem 2012 9 10704 10.1039/c2jm31232h

[B7] Ikejiri K Kitauchi Y Tomioka K Motohisa J Fukui T Zinc blende and wurtzite crystal phase mixing and transition in indium phosphide nanowires Nano Lett 2011 9 4314 4318 10.1021/nl202365q 21875079

[B8] Wang J Plissard SR Verheijen MA Feiner LF Cavalli A Bakkers EP Reversible switching of InP nanowire growth direction by catalyst engineering Nano Lett 2013 9 3802 3806 10.1021/nl401767b 23898831

[B9] Dick KA Caroff P Bolinsson J Messing ME Johansson J Deppert K Wallenberg LR Samuelson L Control of III–V nanowire crystal structure by growth parameter tuning Semicond Sci Tech 2010 9 024009 10.1088/0268-1242/25/2/024009

[B10] Glas F Harmand JC Patriarche G Why does wurtzite form in nanowires of III-V zinc blende semiconductors? Phys Rev Lett 2007 9 146101 17930689 10.1103/PhysRevLett.99.146101

[B11] Kitauchi Y Kobayashi Y Tomioka K Hara S Hiruma K Fukui T Motohisa J Structural transition in indium phosphide nanowires Nano Lett 2010 9 1699 1703 10.1021/nl1000407 20387797

[B12] Hou JJ Han N Wang F Xiu F Yip S Hui AT Hung T Ho JC Synthesis and characterizations of ternary InGaAs nanowires by a two-step growth method for high-performance electronic devices ACS Nano 2012 9 3624 3630 10.1021/nn300966j 22443352

[B13] Han N Wang F Hui AT Hou JJ Shan GC Fei X Hung TF Ho JC Facile synthesis and growth mechanism of Ni-catalyzed GaAs nanowires on non-crystalline substrates Nanotechnology 2011 9 285607 10.1088/0957-4484/22/28/285607 21654028

[B14] Tian B Xie P Kempa TJ Bell DC Lieber CM Single-crystalline kinked semiconductor nanowire superstructures Nat Nanotechnol 2009 9 824 829 10.1038/nnano.2009.304 19893521 PMC2789864

[B15] Krishnamachari U Borgstrom M Ohlsson BJ Panev N Samuelson L Seifert W Larsson MW Wallenberg LR Defect-free InP nanowires grown in [001] direction on InP (001) Appl Phys Lett 2004 9 2077 10.1063/1.1784548

[B16] Wang X Ding Y Summers CJ Wang ZL Large-scale synthesis of six-nanometer-wide ZnO nanobelts J Phys Chem B 2004 9 8773 8777 10.1021/jp048482e

[B17] Marks L Smith DJ HREM and STEM of defects in multiply-twinned particles J Microsc 1983 9 249 261 10.1111/j.1365-2818.1983.tb04222.x

[B18] Hurle D Rudolph P A brief history of defect formation, segregation, faceting, and twinning in melt-grown semiconductors J Cryst Growth 2004 9 550 564 10.1016/j.jcrysgro.2003.12.035

[B19] Korgel BA Semiconductor nanowires: twins cause kinks Nat Mater 2006 9 521 522 10.1038/nmat1688 16819474

[B20] Algra RE Verheijen MA Borgstrom MT Feiner LF Immink G Van Enckevort WJ Vlieg E Bakkers EP Twinning superlattices in indium phosphide nanowires Nature 2008 9 369 372 10.1038/nature07570 19020617

[B21] Wang C Wei Y Jiang H Sun S Bending nanowire growth in solution by mechanical disturbance Nano Lett 2010 9 2121 2125 10.1021/nl100661v 20499890

[B22] Cao AJ Wei YG Mao SX Deformation mechanisms of face-centered-cubic metal nanowires with twin boundaries Appl Phys Lett 2007 9 151909 10.1063/1.2721367

